# The Late Sir Henry Burdett

**Published:** 1920-05-15

**Authors:** Adeline E. Cable

**Affiliations:** *Matron.* Salisbury Infirmary


### THE EDITOR'S LETTER-BOX.

**Correspondence on all subjects is invited, but we cannot in any way be responsible for the opinions expressed by our correspondents, who must give their name and address as a guarantee of good faith, but not necessarily for publication. Correspondents are reminded that brevity of style and conciseness of statement greatly facilitate early insertion.**

### The Late Sir Henry Burdett.

*To the Editor of* The Hospital.

Sir,—Will you allow me to express to you and The Hospital staff my sincere sympathy in the great sorrow that has fallen upon you by the loss of your "Editor-in-chief." Sir Henry did so much for nurses that everywhere they will feel that a strong friend has been taken from them. I have the memory of a visit to our hospital, and a long and helpful talk, as well as kind communications at other times.

Trusting you will have strength for the difficult task of carrying on the great work, I am, Yours sincerely,

Adeline E. Cable,
*Matron.*

Salisbury Infirmary,
May 8, 1920.

